# Use it or lose it: gene–environment interactions at the nexus of expanding genes and shrinking brains in Huntington’s disease and other experience-dependent disorders of ageing

**DOI:** 10.1093/braincomms/fcac308

**Published:** 2022-11-24

**Authors:** Anthony J Hannan

**Affiliations:** Florey Institute of Neuroscience and Mental Health, University of Melbourne, Parkville, VIC 3010, Australia; Department of Anatomy and Physiology, University of Melbourne, Parkville, VIC 3010, Australia

## Abstract

This scientific commentary refers to ‘Intellectual enrichment and genetic modifiers of cognition and brain volume in Huntington’s disease’ by Papoutsi *et al*. (https://doi.org/10.1093/braincomms/fcac279).

**This scientific commentary refers to ‘Intellectual enrichment and genetic modifiers of cognition and brain volume in Huntington’s disease’ by Papoutsi *et al*. (https://doi.org/10.1093/braincomms/fcac279).**

Huntington’s disease is one of the most extraordinary, and devastating, of all human disorders. These exceptional aspects of Huntington’s disease include genetics, with Huntington’s disease being one of the first diseases discovered (three decades ago) to be caused by a tandem-repeat mutation, or ‘genetic stutter’. The monogenic autosomal-dominant nature of Huntington’s disease, associated with the cytosine-adenine-guanine (CAG) trinucleotide repeat encoding an expanded polyglutamine tract in the huntingtin protein, means that the children of those with the gene mutation face the ‘flip of a conceptual coin’, affecting whether they will suffer from this currently incurable disease (after receiving the tandem-repeat expanded Huntington’s disease allele from one of their parents) or escape such a dreadful fate. Other striking aspects of Huntington’s disease include its complex combination of neurological, psychiatric and peripheral symptoms. Whilst the cognitive deficits (culminating in dementia), psychiatric symptoms (the most common of which is depression) and movement disorder (including chorea) are well known, the pervasive peripheral symptoms (such as gastrointestinal dysfunction) are often overlooked.

Huntington’s disease had long been called ‘the epitome of genetic determinism’, listed in textbooks and lectures as ‘100% genetic’, and the exemplar of ‘nature over nurture’. However, all of this changed at the turn of this millennium, when it was reported in a transgenic mouse model of Huntington’s disease that environmental enrichment (which increases environmental novelty and complexity so as to enhance sensory stimulation, cognitive activity and physical exercise) could delay the onset of disease in these Huntington’s disease mice.^1^ This was followed by translation in clinical studies, where a role for environmental modulation of Huntington’s disease pathogenesis was clearly demonstrated,^2,3^ and a range of preclinical and clinical studies in other disorders.^4^ However, these early clinical studies^2,3^ could only show that environmental modifiers affected the onset of clinical (motor) symptoms but could not provide much insight into exactly which environmental factors and exposures, and associated lifestyle parameters, were modulating Huntington’s disease onset (and progression).

An important new study by Papoutsi *et al*.,^5^ in *Brain Communications*, provides novel insights into genetic and environmental modifiers, and associated gene-environment (G x E) interactions, in Huntington’s disease. These investigators used Track-HD, a powerful longitudinal clinical study of Huntington’s disease gene mutation carriers across multiple international centres, to explore potential genetic and environmental contributions to Huntington’s disease pathogenesis.^5^ The key lifestyle factor found to affect Huntington’s disease onset was intellectual enrichment, which was defined as being higher in ‘those who were better educated, had higher verbal intelligence and performed an occupation that was intellectually engaging’.^5^ The subgroup with greater intellectual enrichment was found to have superior overall cognitive function, consistent with previous studies involving Huntington’s disease and other dementias.^6^

Furthermore, intellectual enrichment (as an environmental factor) was found to interact with genetics via a well-studied polymorphism (Val66Met) in the brain-derived neurotrophic factor (*BDNF*) gene.^5^ This link to BDNF is very interesting, as both the Huntington’s disease mutation and specific relevant environmental factors (including cognitive enrichment and physical activity) have been found to modulate BDNF expression in a transgenic mouse model of Huntington’s disease.^7,8^

Finally, this study also examined the potential role of a number of gene candidates as genetic modifiers in Huntington’s disease.^5^ These investigators found that a previously studied polymorphism in the *MSH3* gene had significant effects on cognitive function and neurodegeneration (measured as volumetric atrophy) in multiple regions of the cerebral cortex.^5^ These findings are consistent with a previously proposed role for *MSH3* as a genetic modifier of Huntington’s disease, via modulation of somatic CAG-repeat stability, and strengthen the link with cognitive deficits, which ultimately culminate in dementia.

The most important findings in this new study by Papoutsi *et al*.^4^ related to the beneficial effects of intellectual enrichment, linking these results to the broader concept of ‘brain and cognitive reserve’ (BCR). Whilst the definition of intellectual enrichment included education, verbal intelligence and intellectual engagement via occupational employment,^5^ other studies have included a range of measures of intellectual enrichment in the context of dementia and other brain disorders, and inferred their relationship to BCR.^6^ BCR may provide neuroprotection (or ‘neuroresilience’) against a wide range of neurodegenerative disorders, including Huntington’s disease, Alzheimer’s disease and Parkinson’s disease, although we are only beginning to understand the relevant mechanisms and therapeutic implications.

There are two major implications of our present understanding of BCR and its relevance to brain disorders. Firstly, BCR has major public-health implications. By increasing BCR at a population level, we might be able to delay the onset of a wide variety of neurological and psychiatric disorders. BCR may not only depend on environmental factors and exposures such as intellectual enrichment and physical activity but could be modulated by additional important lifestyle factors such as diet and stress.^4^ Therefore, public-health initiatives that encourage and facilitate healthier lifestyles across the lifespan (including childhood, adolescence and adulthood) could have major impacts, at a population level, in delaying the onset of dementia (e.g. Alzheimer’s disease) and other brain disorders.

Another major implication of such findings is that we urgently need to understand the mechanisms mediating BCR, which would have relevance for the development of novel approaches to prevent and treat a wide variety of brain disorders (Fig. 1). This knowledge would allow us to establish new prophylactic and therapeutic interventions to delay and ameliorate brain disorders, including enviromimetics.^9^ Enviromimetics are therapeutics that mimic or enhance the beneficial effects of environmental stimulation, including cognitive activity and physical exercise.^9^ Intriguingly, the present study^5^ identifies one of the key molecular systems that has previously been considered as a potential target of enviromimetics, BDNF and its associated receptors and downstream signalling pathways.^9^ However, we know that the molecular and cellular effects of cognitive stimulation and physical activity are extensive and diverse.

**Figure 1:**
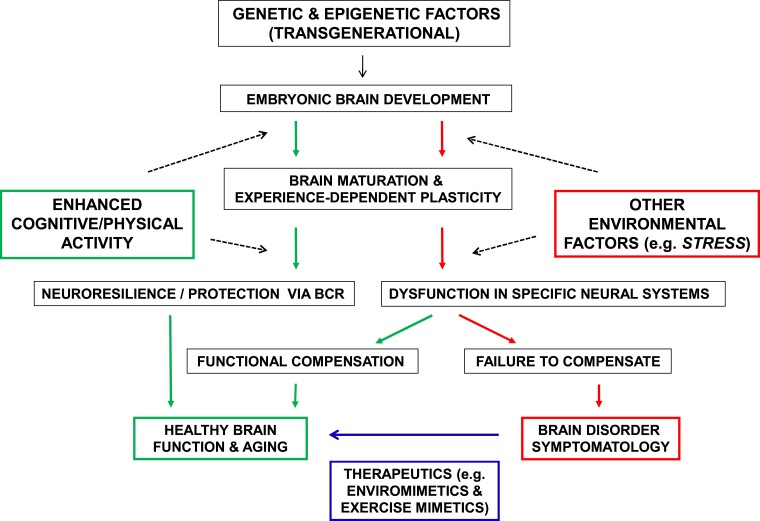
Schematic illustration of the contributions of genetic and environmental factors, and BCR, to the pathogenesis of brain disorders. Each individual commences life at conception, receiving a genome and epigenome (via intergenerational epigenetic inheritance) from their parents. Throughout *in utero* development, environmental factors begin to interact with genetic and epigenetic factors to sculpt brain maturation, and this continues postnatally and throughout adult life. Some individuals, due a combination of genetic, epigenetic and environmental factors, have a trajectory (indicated by red arrows, on the right-hand side) associated with brain dysfunction. Beneficial environmental factors such as enhanced cognitive and physical activity can help build neuroprotection and resilience via BCR and preserve a trajectory of healthy brain function (indicated by green arrows, on the left-hand side). These beneficial environmental factors may lead to functional compensation in a dysfunctional brain and thus a shift towards the trajectory of healthy brain function. Detrimental environmental factors (such as increased levels of chronic stress) can trigger or exacerbate brain dysfunction. A subset of individuals will develop a specific brain disorder, reflecting the effects of genetic, epigenetic and environmental factors overlaid on brain ageing, as well as stochastic biological processes. Those who develop a brain disorder require targeted therapeutics informed by precision-medicine approaches, such as enviromimetics, and their subclass, exercise mimetics.

The cellular mediators of BCR include aspects of neuronal structure and function, including numbers of specific neuronal and synaptic subtypes, neuronal morphology and physiology. However, the human brain is the most complex organic structure in the known universe and we know that the ∼100 billion neurons are accompanied by at least as many glial cells, which are all fed by a vast and complex neurovascular system. The complexity does not end there, as many of the trillions of host cells in the human body are in direct (or indirect) communication with the brain, along with many trillions of microbes (mainly bacteria) that occupy the body (mainly the gastrointestinal system). Thus, in considering BCR, we must also consider the structure and function of neurons, glia and the many other cells which reside in, or interact with, the brain.

This key issue of brain–body interactions associated with BCR has been exemplified in studies of Huntington’s disease. The Huntington’s disease gene and its encoded protein (huntingtin) are expressed throughout the brain and body, from development onwards. Therefore, BCR in Huntington’s disease may be affected not only by changes to neurons, glia and other cells within the brain but also by changes in various biological systems (and their constituent cells) that occur outside the brain but are linked via dynamic bidirectional brain–body signalling. This complex brain–body signalling can include molecules delivered via the bloodstream (including those associated with metabolic, endocrine and immune function) that may have their origin in a diverse range of organs and tissues, including both human host cells and the microbes that inhabit our gastrointestinal and other systems.

So, how might we harness the therapeutic effects of cognitive stimulation and physical activity, and other environmental stimuli associated with BCR, to prevent and treat brain disorders? A path forward has recently been proposed for exercise mimetics, which is a subclass of enviromimetics.^10^ The starting point needs to be a sophisticated understanding of BCR, at molecular, cellular, circuit and systems levels. Whilst the initial targets of enviromimetics will be most likely be molecular, sophisticated screening processes and platforms are required to determine which molecular and cellular changes are necessary or sufficient for the beneficial effects of BCR.

In conclusion, this new study^5^ adds important pieces to the puzzle of pathogenesis in Huntington’s disease, in particular the role of genetic and environmental modifiers. This knowledge will be important in the ongoing attempts to develop novel approaches to prevent and treat Huntington’s disease. Recent failures of the first clinical trials involving huntingtin-lowering approaches to Huntington’s disease emphasize the need to keep all therapeutic options on the table, including relatively new concepts such as enviromimetics and exercise mimetics (Fig. 1).

However, this expanding field of research has implications far beyond Huntington’s disease. For every disorder of ageing, including neurodegenerative diseases such as Huntington’s disease, Alzheimer’s disease and Parkinson’s disease, the greatest risk factor is age. Genetic and environmental factors interact and can be overlaid on ageing, to ‘push or pull’ the dynamic trajectory of healthy maturation and ageing towards, or away from, dysfunctional and diseased states (Fig. 1). The deliberate simplicity of this diagrammatic schema (Fig. 1) masks the enormous complexity of molecular, cellular, circuit and systems dynamics over time. Rather than fleeing from such complexity (for the comfort of reductionist simplicity), we as neuroscientists and medical researchers must increasingly learn to confront the complexity, armed with a powerful arsenal of 21st-century science and technology. By taking on these grand challenges of neuroscience and medicine, we will provide hope and new clinical options for the millions of families around the world that are threatened by these devastating disorders of the brain, and mind.

## Data Availability

Data sharing is not applicable to this article as no new data were created or analysed.
